# Study of Combined Effect of Bacteriophage vB3530 and Chlorhexidine on the Inactivation of *Pseudomonas aeruginosa*

**DOI:** 10.1186/s12866-023-02976-w

**Published:** 2023-09-13

**Authors:** Yan Liu, Yining Zhao, Changrui Qian, Zeyu Huang, Luozhu Feng, Lijiang Chen, Zhuocheng Yao, Chunquan Xu, Jianzhong Ye, Tieli Zhou

**Affiliations:** 1 Department of Clinical Laboratory, Key Laboratory of Clinical Laboratory Diagnosis and Translational Research of Zhejiang Province, the First Affiliated Hospital of Wenzhou Medical University, Wenzhou, 325000 China; 2 School of Basic Medical Sciences, Wenzhou Medical University, Wenzhou, 325000 Zhejiang Province China; 3 Department of Medical Lab Science, School of Laboratory Medicine and Life Science, Wenzhou Medical University, Wenzhou, Zhejiang Province China

**Keywords:** Bacteriophages, Chlorhexidine, Chlorhexidine tolerance, Synergistic effect, *Pseudomonas aeruginosa*

## Abstract

**Background:**

Chlorhexidine (CHG) is a disinfectant commonly used in hospitals. However, it has been reported that the excessive use of CHG can cause resistance in bacteria to this agent and even to other clinical antibiotics. Therefore, new methods are needed to alleviate the development of CHG tolerance and reduce its dosage. This study aimed to explore the synergistic effects of CHG in combination with bacteriophage against CHG-tolerant *Pseudomonas aeruginosa* (*P. aeruginosa*) and provide ideas for optimizing disinfection strategies in clinical environments as well as for the efficient use of disinfectants.

**Methods:**

The CHG-tolerant *P. aeruginosa* strains were isolated from the First Affiliated Hospital of Wenzhou Medical University in China. The bacteriophage vB3530 was isolated from the sewage inlet of the hospital, and its genome was sequenced. Time-killing curve was used to determine the antibacterial effects of vB3530 and chlorohexidine gluconate (CHG). The phage sensitivity to 16 CHG-tolerant *P. aeruginosa* strains and PAO1 strain was detected using plaque assay. The emergence rate of resistant bacterial strains was detected to determine the development of phage-resistant and CHG-tolerant strains. Finally, the disinfection effects of the disinfectant and phage combination on the surface of the medical devices were preliminarily evaluated.

**Results:**

The results showed that (1) CHG combined with bacteriophage vB3530 significantly inhibited the growth of CHG-resistant *P. aeruginosa* and reduced the bacterial colony forming units (CFUs) after 24 h. (2) The combination of CHG and bacteriophage inhibited the emergence of phage-resistant and CHG-tolerant strains. (3) The combination of CHG and bacteriophage significantly reduced the bacterial load on the surface of medical devices.

**Conclusions:**

In this study, the combination of bacteriophage vB3530 and CHG presented a combined inactivation effect to CHG-tolerant *P. aeruginosa* and reduced the emergence of strains resistant to CHG and phage. This study demonstrated the potential of bacteriophage as adjuvants to traditional disinfectants. The use of bacteriophage in combination with commercial disinfectants might be a promising method for controlling the spread of bacteria in hospitals.

## Background

*Pseudomonas aeruginosa* (*P. aeruginosa*) is a gram-negative opportunistic bacterial pathogen, causing infections in immunocompromised patients [1, 2]. It can spread rapidly through various channels, such as tap water, cleaning equipment, oral swabs, etc. [3–5]. Multidrug resistance in *P. aeruginosa* is common and mortality from invasive infections is as high as 29% [6]. Therefore, it is important to control the spread of this organism.

Chlorhexidine (CHG) is a biguanide topical antiseptic agent with broad antibacterial activities. It is widely used in hospital intensive care unit patient care, skin cleaning and medical device disinfection [7–10]. Although this disinfectant has a good bactericidal effect, its widespread use can lead to a gradual increase in bacterial resistance to CHG and cross-resistance to other traditional antimicrobial agents [11]. Lu Ji et al. also showed that chemical disinfectants lead to the survival of only antimicrobial-resistant strains, which can then spread throughout the population by various means [12]. In addition, related studies have also shown that excessive residual chlorine can damage the aquatic environment, exerting toxic effects on various aquatic organisms [13]. Therefore, studies are urgently required to explore ways for reducing the amount of CHG usage as an antibacterial agent.

Bacteriophages are a class of viruses that can kill bacteria. They infect bacteria through different stages, including adsorption, injection, replication, transcription and translation, assembly, and release. They use bacterial machinery to replicate and reproduce, which leads to bacterial cell lysis and death [14]. In recent years, phage-based products have gradually attracted the attention of researchers due to their advantages, such as their high specificity to bacteria, strong lysis ability, no toxic side effects on humans and animals (products), no drug residue [15], and effective elimination or reduction bacterial contamination [16]. Hoopes J Todd et al. developed the first bacteriophage-based disinfectant against *Streptococcus* [17]. Sukumaran Anuraj T et al. found that the combination of bacteriophage with peracetic acid could significantly reduce the population of *Salmonella* [18]. Joshua P et al. reported that the combination of bacteriophage cocktail and levulinic acid lotion showed a better antibacterial effect on the food-borne pathogens *Escherichia coli* O157:H7, *Shigella*, and *Salmonella* on the surface of vegetables [19]. In fact, several bacteriophage-based products have been developed and are commercially available. For instance, the products containing anti-*Escherichia coli* O157:H7 and *Salmonella* bacteriophages produced by Intralytix have also been approved by the United States Food and Drug Administration (FDA) [20].

Facing the situation of resistance in chemical disinfectants such as CHG, safer biological disinfection agents with low toxicity and high efficiency are urgently needed to replace traditional disinfectants. Therefore, the current study investigated the bacteriostatic effects of combining bacteriophage with CHG against CHG-tolerant *P. aeruginosa* and measured the frequency of CHG-tolerant and phage-resistant strains of *P. aeruginosa*. Moreover, the combined antibacterial effects of CHG and bacteriophage on the surface of medical devices were also evaluated.

## Results

### Isolation and morphology of *P. aeruginosa* bacteriophage vB3530

**Fig. 1:**
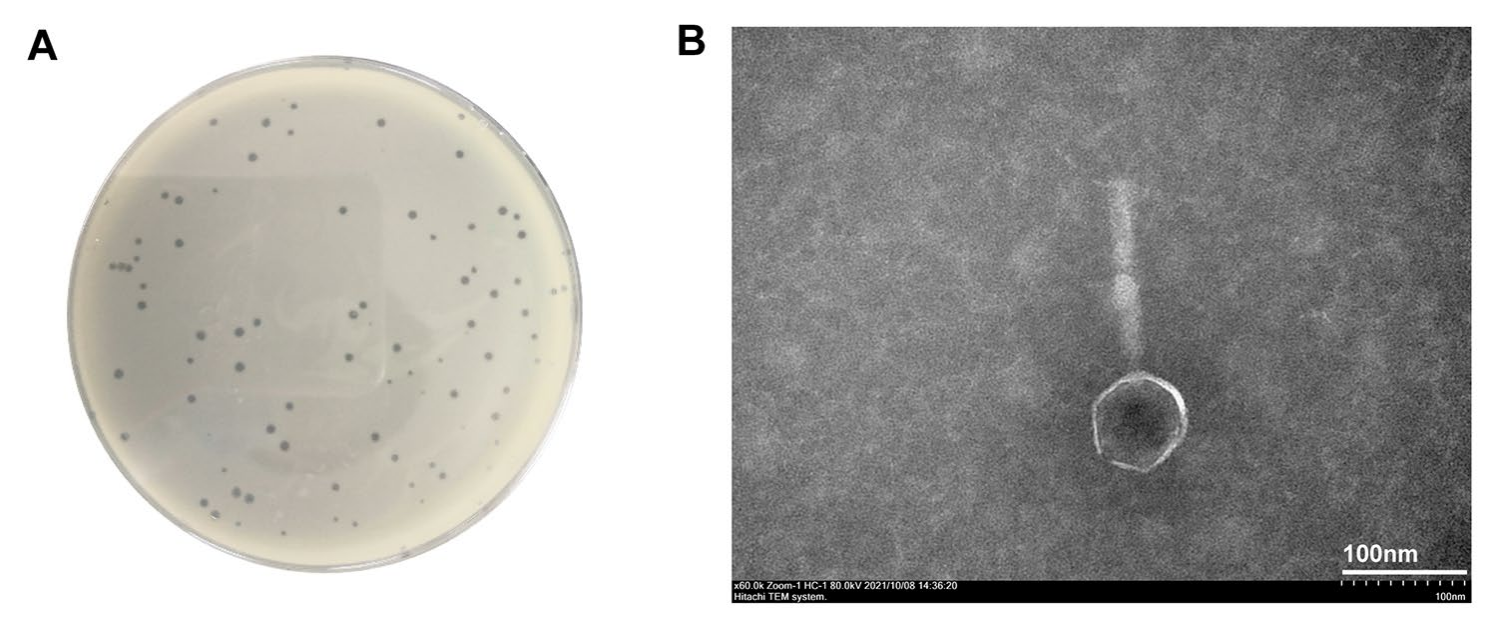
The plaque (**A**) and the TEM morphology (**B**) of bacteriophage vB3530

Clinical CHG-tolerant *P. aeruginosa* strains were used as the host strains to test the sensitivity of bacteriophage vB3530 (Table 1). The vB3530 formed minor plaques of about 1 mm diameter on double layer ager (Fig. 1A). The transmission electron microscopy (TEM) observation showed that vB3530 had a head of 73 nm in diameter and a tail of 162 nm long and 15 nm wide, belonging to the *Caudovirales* order of viruses (Fig. 1B).

**Table 1:** Sequence types (ST), clinical sample source of chlorhexidine-tolerant *P. aeruginosa* isolates and host range and efficiency of plating (EOP) of phage vB3530 determined against 16 strains. Clear lysis zone (+) and not lysis zone (−)

Strain	ST	Sample	Host range	EOP
TL3569	ST381	Sputum	**+**	2.5 × 10^− 1^
TL3649	ST856	Urine	-	0
TL3651	ST2449	Sputum	**+**	6.0 × 10^− 1^
TL3652	ST1968	ICU	**+**	7.2 × 10^− 1^
TL3670	ST277	Sputum	**+**	5.5 × 10^− 1^
TL3674	ST274	Sputum	**+**	0
TL3683	ST644	ICU	**+**	0
TL3692	ST1968	Drainage	**+**	0
TL3706	ST644	ICU	**+**	1.8 × 10^− 2^
TL3727	ST1249	Sputum	-	0
TL3733	ST980	ICU	**+**	0
TL3761	ST242	ICU	**+**	0
TL3763	ST377	Blood	-	0
TL3767	ST377	Pus	**+**	4.8 × 10^− 1^
TL3780	ST557	Emergency	**+**	0
TL3783	ST463	ICU	**+**	0

### Genome characteristics of *P. aeruginosa* bacteriophage vB3530

**Fig. 2:**
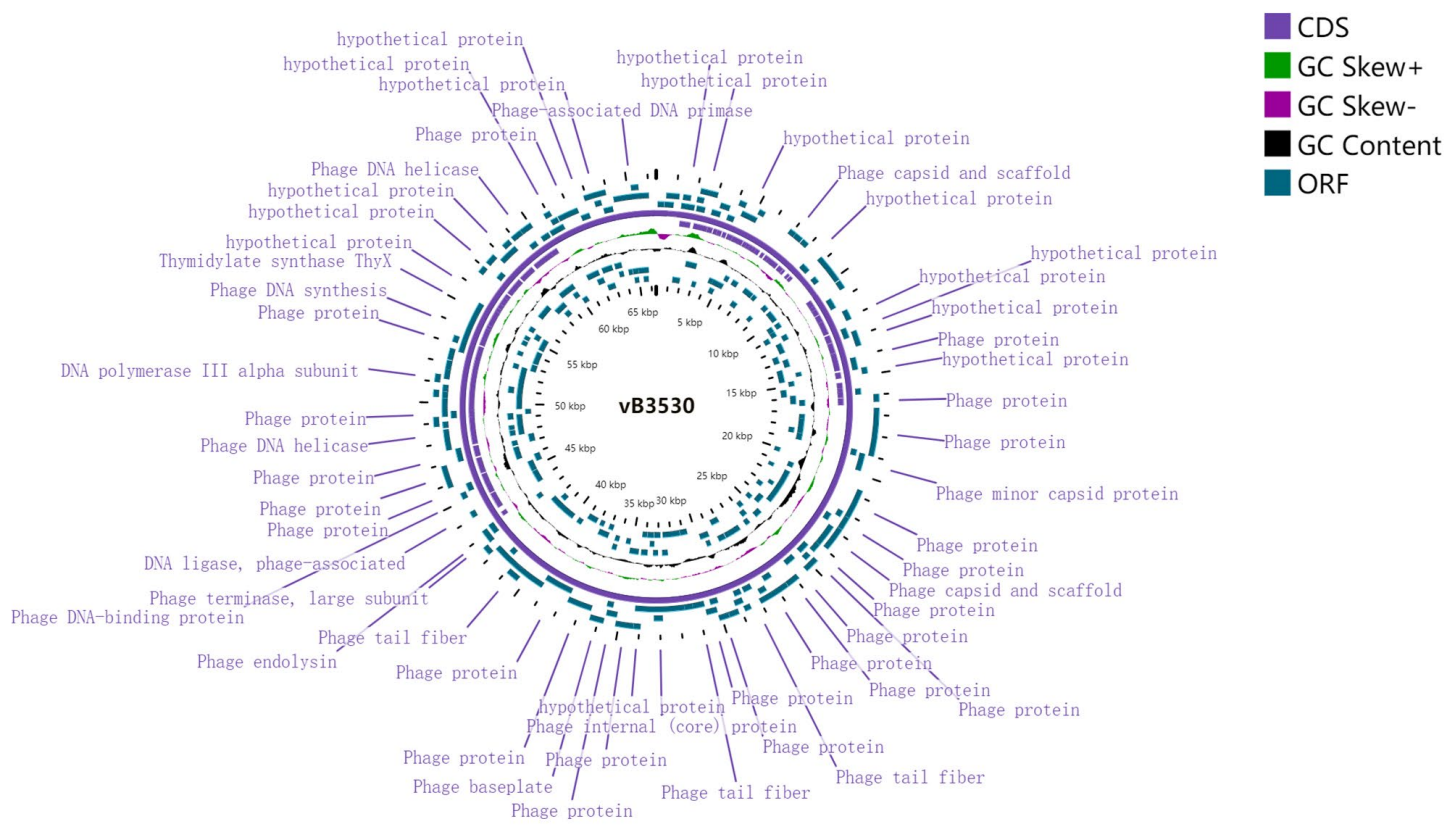
The genome annotation of bacteriophage vB3530

Phage vB3530 had a double-stranded genome, which was 66,392 base pairs (bps) long with a GC content of 55.65% and contained 95 open reading frames (ORFs), as annotated by DNA master software (Fig. 2). The genome was also analyzed using the Comprehensive Antibiotic Resistance Database (CARD) and the Virulence Factors of Pathogenic Bacteria (VFPB) database, and the results showed that vB3530 had no antibiotic resistance genes (ARGs) or virulence genes. According to the whole genome sequence analysis, vB3530 was similar to *Pbunavirus*, a member of the *Myoviridae* family (Fig. 2). The complete genome sequence of phage vB3530 was deposited in GenBank under the accession number OR075999.

### Sensitivity and efficiency of plating (EOP) analysis of phage vB3530

**Fig. 3:**
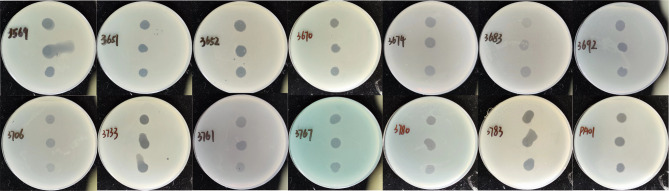
Phage sensitivity assay. Clear plaques formed by 10-fold serial dilutions of phage vB3530 on the lawns of 13 CHG-tolerant strains and reference strain PAO1

These 16 isolates were divided into 13 sequence types (STs) using multi-locus sequence typing (MLST) analysis (Table 1), demonstrating that the strains were not genetically related clones. Host profiling experiments for vB3530 were then performed using 16 CHG-tolerant *P. aeruginosa* strains and reference strain PAO1. In host range spotting tests, phage vB3530 showed lytic activity against 13 of 16 CHG-tolerant *P. aeruginosa* strains and formed transparent lytic spots on the top layer of the double agar plate, thereby showing a wide host range (Fig. 3). These results indicated that the bacteriophage vB3530 could lyse the host bacteria regardless of the ST type. The results of EOP experiments showed the highest lysis efficiency of vB3530 for TL3652 (Table 1).

### Minimum inhibitory concentration (MIC) of CHG and antibiotics against six strains of CHG-tolerant *P. aeruginosa*

The MIC values of CHG and different classes of antibiotics against six CHG-tolerant *P. aeruginosa* strains, which could be lysed by vB3530 were tested using the microbroth dilution method. Among the tested strains, only two strains, including TL3652 and TL3670, were resistant to cephalosporins (Table 2). The MIC value of CHG against other strains was 64 µg/mL except for TL3706, for which the MIC value was 32 µg/mL. The MIC of CHG against the reference strain was also PAO1tested, showing the MIC value of 16 µg/mL.

**Table 2:** Minimum inhibitory concentrations (mg/L) of chlorhexidine and other antibacterial agents against chlorhexidine-tolerant strains. ^A^Bold values point means resistance. Abbreviations: CAZ, Ceftazidime; IMP, Imipenem; CIP, Ciprofloxacin; GEN, Gentamicin; CHG, Chlorhexidine

Strains	Cephalosporins	Carbapenems	Fluoroquinolone	Aminoglycosides	Sanitizer	CHG-resistant mechanism
	CAZ	IPM	CIP	GEN	CHG	
TL3569	8	2	0.25	0.125	**64**	Unknown
TL3651	2	0.5	0.25	0.25	**64**	*cepA*
TL3652	**256**	32	1	2	**64**	*cepA*
TL3670	**256**	2	0.5	0.25	**64**	*cepA*
TL3706	4	2	0.125	0.5	32	Unknown
TL3767	4	4	0.25	0.125	**64**	*cepA*

### Time-killing curves of sub-inhibitory concentrations (sub-MICs) of CHG combined with vB3530 against CHG-tolerant strains

**Fig. 4:**
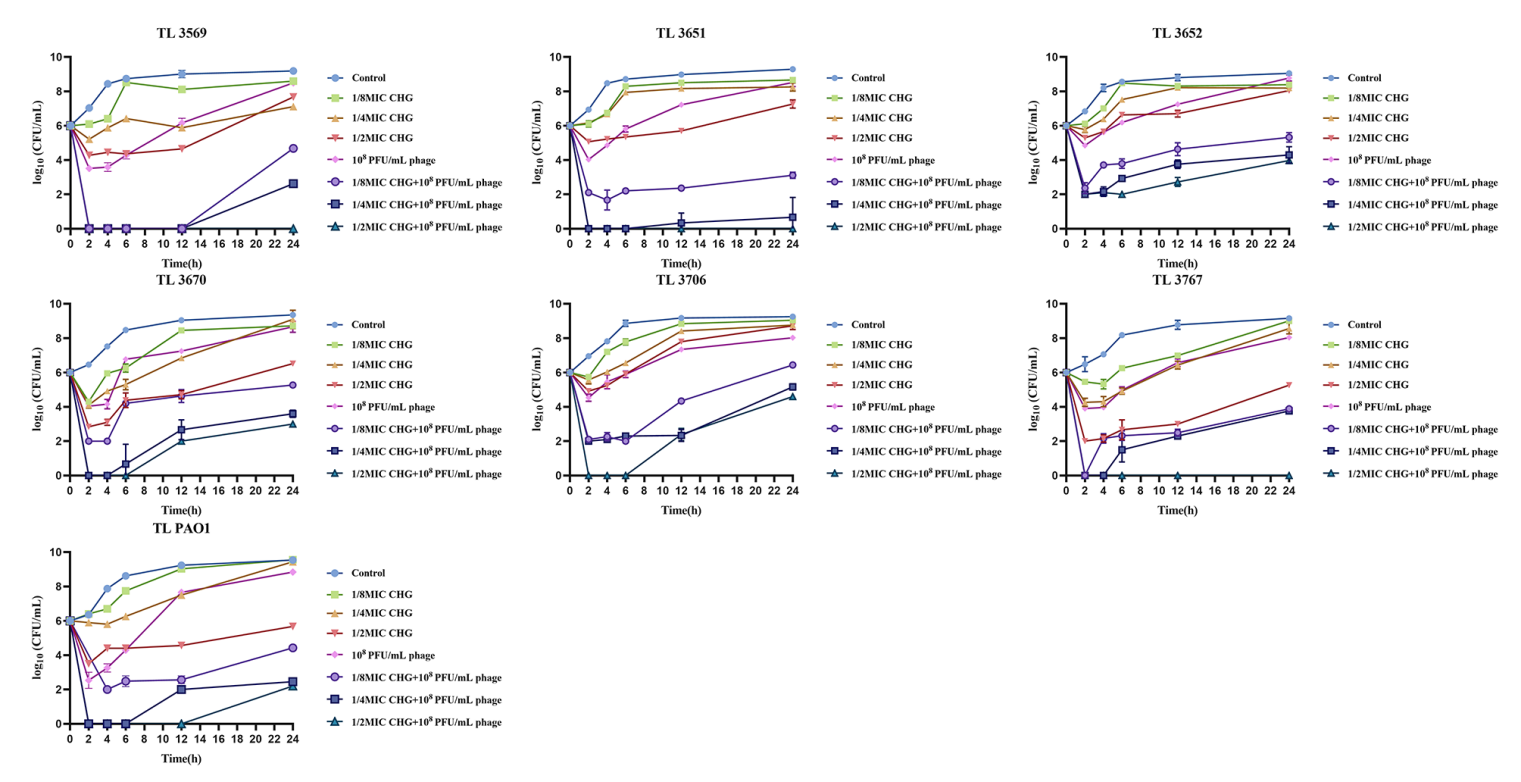
Time-killing curves of CHG alone and in combination with phage vB3530 against CHG-tolerant *P. aeruginosa* and reference strain PAO1

In the time-killing curves, phage or CHG at sub-MIC showed no bactericidal activity over the 24-h period, and the bacterial colony forming units (CFUs) in phage or CHG monotherapy group exceeded the initial CFUs (6 log_10_ CFU/mL) after 24 h, except that the CFUs of the 1/2×MIC CHG group in TL 3767 and TL PAO1 were lower than the initial CFUs within 24 h (5.26 and 5.68 log_10_ CFU/mL, respectively). In contrast, compared to phage or CHG monotherapy, the treatment with the phage/CHG combination at the same concentration as the monotherapy resulted in a significant reduction in CFUs after 24 h. And except for the 1/8×MIC CHG + phage group in TL3706, the CFUs in the combined group of other strains were all lower than the initial CFUs at 24 h (Fig. 4). In addition, it can be seen from the figure that the combination of vB3530 and CHG exhibited the best effect in the 1/2×MIC CHG + 1 × 10^8^ PFU/mL phage group, especially against TL3569 and TL3767 strains. In conclusion, these results suggested that the combination of bacteriophage and CHG played a significant inactivation effect against CHG-tolerant *P. aeruginosa* strains, but the effect varied among different strains.

### Determination of the emergence rate of bacterial resistant mutants

**Fig. 5:**
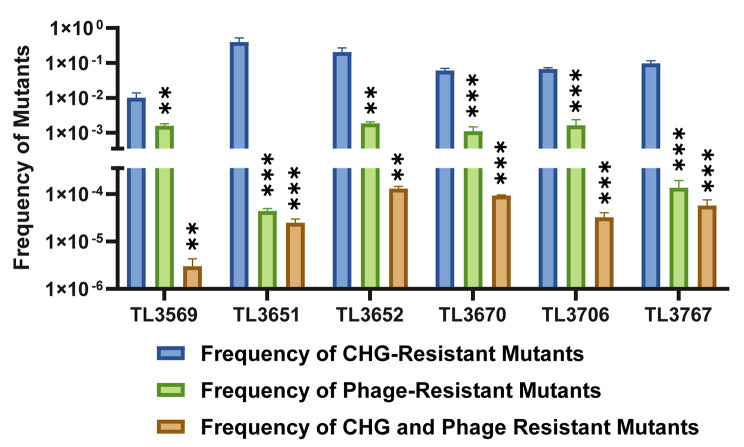
Frequency of bacterial phage-resistant and CHG-tolerant strains treated with CHG or phage alone and in combination

The results showed that the frequency of resistant *P. aeruginosa* strains was different when treated with either the combination of CHG and bacteriophage or individually. As shown in Fig. 5; Table 3, when *P. aeruginosa* was treated with the combination of CHG and phage, the frequency of resistant strains was much lower than those of bacteriophage or CHG alone (*P* < 0.05). Besides, the resistant TL3569 strain (3.01 ± 1.30 × 10^− 6^) had the lowest frequency among all strains when treated with the combination of 1/2×MIC CHG and phage. However, under the same treatment conditions, the frequency of the resistant TL3652 strain (1.31 ± 0.14 × 10^− 4^) was significantly higher than those of the other strains. In conclusion, the combination of CHG and phage could simultaneously reduce the frequency of CHG-tolerant and phage-resistant strains.

**Table 3:** Frequency of *P. aeruginosa* chlorhexidine-tolerant and phage-resistant strains in different treatments

Strain	1/2 MIC CHG Treatment	Phage Treatment	1/2 MIC CHG and phage Treatment
	Frequency of CHG-Resistant Mutants	Frequency of Phage-Resistant Mutants	Frequency of CHG and Phage Resistant Mutants
TL3569	1.01 ± 0.38 × 10^− 2^	1.58 ± 0.23 × 10^− 3^	3.01 ± 1.30 × 10^− 6^
TL3651	2.48 ± 1.90 × 10^− 1^	4.38 ± 0.58 × 10^− 5^	2.48 ± 0.49 × 10^− 5^
TL3652	2.06 ± 0.64 × 10^− 1^	1.86 ± 0.21 × 10^− 3^	1.31 ± 0.14 × 10^− 4^
TL3670	6.09 ± 0.94 × 10^− 2^	1.11 ± 0.37 × 10^− 3^	9.27 ± 0.38 × 10^− 5^
TL3706	6.72 ± 0.57 × 10^− 2^	1.64 ± 0.73 × 10^− 3^	3.24 ± 0.79 × 10^− 5^
TL3767	9.66 ± 1.86 × 10^− 2^	1.36 ± 0.58 × 10^− 4^	5.73 ± 1.84 × 10^− 5^

### Combination of CHG and vB3530 effectively eliminated bacteria on the surface of the contaminated needles

**Fig. 6:**
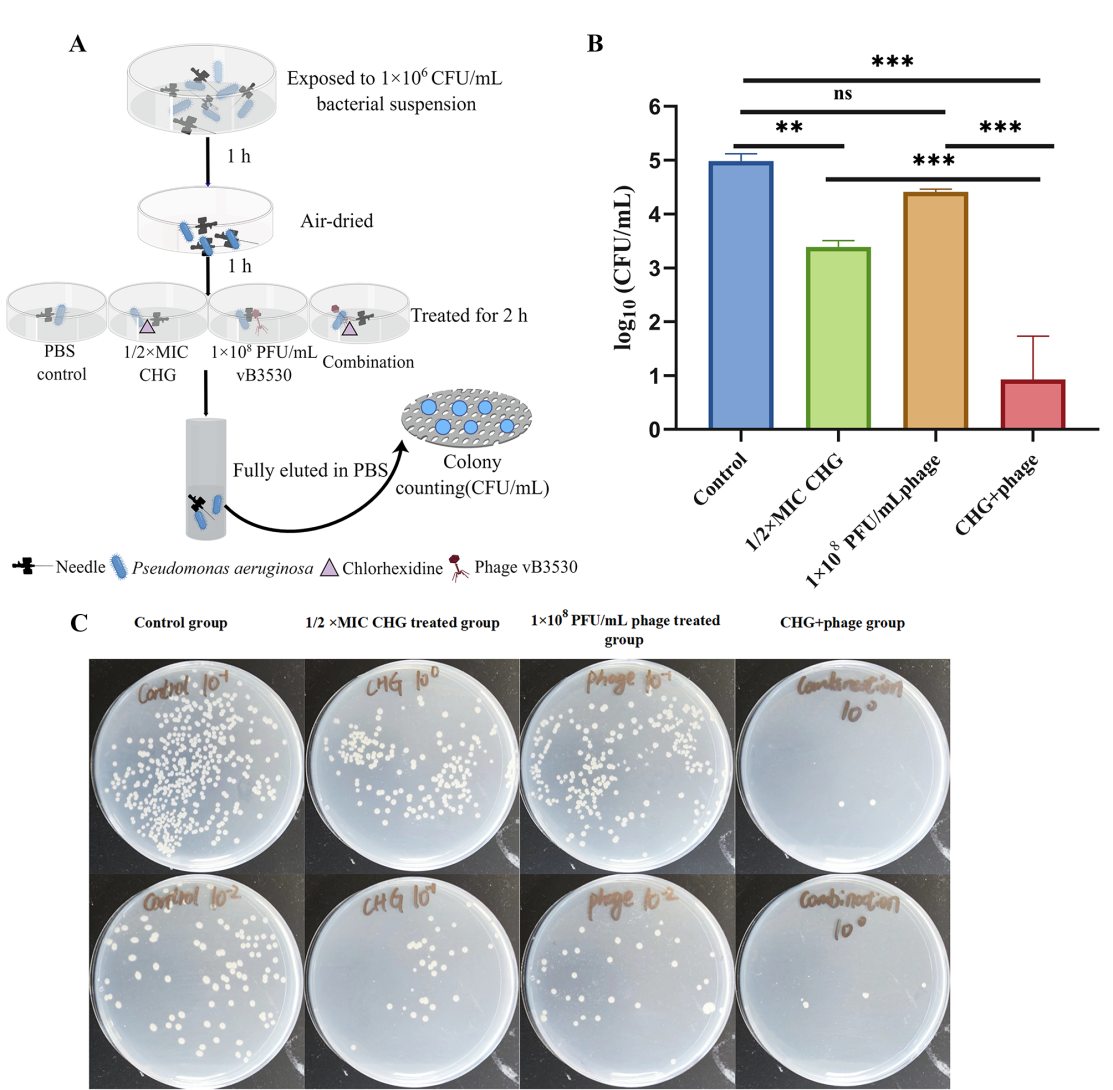
Effects of vB3530 and CHG combination on the removal of bacteria attached to the surface of medical devices. (**A**) Procedures of medical device surface disinfection; (**B-C**) Colony counting. 10^0^, 100 µL of original eluate; 10^− 1^, 100 µL solution diluted 10-fold;10^− 2^, 100 µL solution diluted 100-fold

Subsequently, the disinfecting effects of the combination of CHG and bacteriophage on the surface of medical devices were assessed using needles exposed to *P. aeruginosa* PAO1. The results showed that as compared to the individual treatments of CHG or phage, their combination significantly reduced the bacterial load on the surface of exposed needles (*P* < 0.001, Fig. 6). Thus, this study demonstrated the potential of this combination for the surface disinfection of medical devices.

## Discussion

Due to the gradual increase in the tolerance of clinically isolated *P. aeruginosa* to CHG [21], new methods are needed to restore bacterial susceptibility to CHG. Numerous studies have reported the use of bacteriophage for controlling the growth of pathogenic bacteria in the environment [22, 23]. Agun S et al. demonstrated a good synergistic antibacterial effect of CHG and *S. aureus*-specific bacteriophage [16]. Therefore, the current study explored the interaction of sub-MIC CHG and bacteriophage vB3530 against *P. aeruginosa*.

In host-range experiments, bacteriophage vB3530 was observed to lyse 13 of 16 CHG-tolerant *P. aeruginosa* strains. It has been reported that the difference in receptors on the surface of host bacteria might affect the efficiency of bacterial lysis by bacteriophage. In this study, the EOP of bacteriophage between different host bacteria in comparison with the maximum titer observed varied widely. At the same time, a high rate of bacterial lysis by bacteriophages results in producing a high concentration of endolysin, which lyses bacteria from the outside, leading to a positive host profile test while lacking plaque in the EOP test [24]. This was consistent with the observation in this study, the bacteriophage vB3530 could only form plaques in 6 of the 16 strains positive for the spotting assay.

Many studies have reported the synergistic bactericidal effects of bacteriophages and traditional antibiotics [25–27]. In these studies, bacteriophage and sub-MICs of antibacterial drugs inhibited bacterial growth within 24 h after acting on pathogenic bacteria. In our study, the combination of bacteriophage and sub-MICs of CHG increased the efficacy of bacterial inactivation as compared to their individual treatments. However, the antibacterial status of the combination group was not the same, this may have to do with the surface receptors of the bacteria. Many bacterial surface components have been identified as bacteriophage recognition receptors, including outer membrane proteins (OmpA and OmpC), lipopolysaccharide (LPS), capsular polysaccharide, etc. [28–31]. Fang et al. also reported that the antibacterial effect of bacteriophage was affected by the bacterial serotype [32]. Some studies proved that the combination of bacteriophage and antibiotics could limit the emergence of resistant bacterial strains [33, 34]. This was consistent with the results in the current study. In the 1/2×MIC CHG and phage combination, the overall emergence rate of resistant bacteria was significantly lower than that observed in the CHG or bacteriophage group alone. However, not all phages can reduce the resistance frequency in combination with antibiotics. For example, the bacteriophage-streptomycin combination group showed a similar resistance rate as compared to that observed in the bacteriophage and streptomycin group alone [35]. Since streptomycin inhibits bacterial protein synthesis, thereby significantly impacting the process of bacteriophage proliferation; this restricted bacteriophage production and further limited the role of bacteriophages in reducing the frequency of bacterial resistance [36].

Finally, the antibacterial efficacy of CHG and phage vB3530 combination in surface disinfection of medical devices was evaluated using infected syringe needles. The results showed that bacteriophage and CHG could effectively remove bacteria from the surface of infected needles. Overall, these results suggested the combination of CHG and bacteriophage as promising biological disinfection agent and provided an idea for the combination of bacteriophage and traditional disinfection agents.

However, in this study, only one phage was used as a supplement to CHG to enhance its antibacterial effect. There are numerous types of bacteriophages, and using only one phage for the test might not be enough to explain the role of the phages as a commonly used disinfectant supplement. Therefore, our future studies will focus on using phages that recognize different receptors on bacterial surfaces or those from different families to compose a phage cocktail with a broader host range. In combination with a disinfectant, this cocktail may do better than a phage or disinfectant alone.

## Conclusions

The results in this study proved that the combination of bacteriophage vB3530 and CHG exhibited obvious inactivation effects against CHG-tolerant *P. aeruginosa*, and greatly reduced the development of bacterial resistance to CHG and bacteriophage. The findings in this study demonstrated the potential of using bacteriophages as adjuvants to traditional disinfectants.

## Materials and methods

### Bacterial strains and growth conditions

In this study, 16 CHG-tolerant *P. aeruginosa* strains were isolated from the First Affiliated Hospital of Wenzhou Medical University, China. PAO1 was used as the reference strain. These strains were all identified using matrix-assisted laser desorption/ionization time-of-flight mass spectrometry (MALDI-TOF/MS; bioMérieux, Lyons, France). The multilocus sequence typing (MLST) and drug-resistant genes were analyzed in our previous study [21]. These isolates were stored in Luria Bertani (LB) media, containing 30% glycerol. Before each assay, a single colony of each strain was inoculated into 30 mL of LB with shaking for 16 h to obtain bacteria in the log phase with a concentration of about 10^8–9^ CFU/mL.

### Antibiotics and reagents

Chlorhexidine gluconate was purchased from Macklin Biochemical Technology Co., Ltd. (Shanghai, China). Antibiotics used in this study were purchased from Wenzhou Kangtai Biological Technology Co., Ltd. (Zhejiang, China), including ceftazidime (CAZ), imipenem (IPM), ciprofloxacin (CIP), and gentamicin (GEN). Solutions and diluents of antibiotics were following the latest Clinical and Laboratory Standards Institute guidelines (CLSI 2022).

### Phage isolation and TEM observation

The bacteriophage vB3530 was isolated from sewage samples using *P. aeruginosa* reference strain PAO1 as host bacteria. The purification process was performed as described previously with minor modifications [37]. Briefly, the sewage sample was filtered through a 0.22-µm filter (Millipore Stericup-GP, 0.22 μm, polyethersulfone filter) to remove bacteria. Then, the LB medium and log-phase bacterial suspension was added to the filtered sewage for overnight culture. The next day, the cultures were centrifuged at 5,000 rpm for 15 min, and the supernatant was filtered through a 0.22-µm filter before dripped on the double-layer agar containing host strains. The plaques formed on the upper layer agar were picked and added to SM buffer (50 mM Tris-HCl, pH 7.5, 100 mM NaCl, and 8 mM MgSO_4_).

The bacteriophage particles were plated on carbon-coated copper grids and dried at room temperature for 25 min. Then, the particles were stained with 2% phosphotungstic acid for 15 s. Finally, the sample was examined under a Hitachi H-7500-TEM (Hitachi, Japan) [38].

### Phage propagation and titer determination

Bacteriophage suspension was prepared from the bacteriophage stocks previously prepared in SM buffer (50 mM Tris-HCl, pH 7.5, 100 mM NaCl, and 8 mM MgSO_4_). Briefly, 200 µL of bacteriophage lysate and 1 mL of 1.5 × 10^8^ CFU/mL *P. aeruginosa* bacterial suspension was added to 19 mL of LB. The suspension was cultured in a shaking incubator overnight at 37℃ and 180 rpm for 24 h. The next day, the cultures were centrifuged at 5,000 rpm for 15 min, and the supernatant was filtered through a 0.22-µm filter (Millipore Stericup-GP, 0.22 μm, polyethersulfone filter) to remove intact bacteria and bacterial debris. The bacteriophage suspensions were stored at 4 ℃ [39].

The bacteriophage titer was determined using the double-layer agar method, and the results were expressed as a plaque-forming units (PFU/mL) as described previously with minor modifications [40]. Briefly, the host strain was suspended in an LB medium and incubated at 37℃ for 18 h. Then, a 100 µL bacterial culture was mixed with 100 µL of the phage solution diluted 10-fold and added to 8 mL of melted 0.4% agar. The mixture was poured onto the surface of a lower plate, containing 1.5% agar. Three replicates of each dilution were made. Bacteriophage titer (PFU/mL) = the number of plaques × 10 × reciprocal of counted dilution.

### Bacteriophage genome sequencing and annotations

The genome of bacteriophage vB3530 was sequenced on the Nursing Illumina Hiseq 2500 platform (~ 1 Gbp/sample). Fastp was used for adapter trimming and quality filtering when demultiplexing the raw reads. ORFs were predicted using Rapid Annotations using Subsystems Technology (RAST) toolkit. The manual predictions were confirmed using the PhageAI tool, which is a platform for determining phage life cycles and taxonomy (https://phage.ai/) (27). Phage-encoded ARGs and virulence factors were identified using the online tools from the CARD (https://card.mcmaster.ca/) and VFPB database (http://www.mgc.ac.cn/VFs/) [41, 42].

### Phage host range: spot test and EOP for vB3530

As described in a previous study, the sensitivity of vB3530 against 16 CHG-tolerant *P. aeruginosa* strains and reference strain PAO1 was tested using phage spot assay [43]. Briefly, *P. aeruginosa* was cultured overnight in a fresh LB medium. Then, 100 µL of the culture was mixed with 5 mL of melted 0.4% agar and LB medium to prepare double-layered agar plates. The phages were then added to a 10-fold gradient dilution in SM buffer. Finally, 3 µL aliquots were spotted onto a plate and incubated at 37 °C for 12 h. The bacterial susceptibility to bacteriophages was determined by in situ lytic clearance zones. Based on the clearance zones, bacteria were divided into two categories, including clear lysis zone (+) and non-lysis zone (-).

The EOP of the bacteria (the appearance of a well-defined zone of lysis) was determined using the double-layer agar plate method and calculated as the PFU/mL of tested strain as described previously for CHG-tolerant *P. aeruginosa* strains [44].

### MIC tests

The CHG-tolerant *P. aeruginosa* strains were stored at -80℃ in 30% glycerol. The stored cultures were refreshed onto Columbia blood agar (CBA) plates and incubated at 37℃ overnight. The MICs of CHG and other commonly used antibiotics, including CAZ, IPM, CIP, and GEN (gradient concentrations from 0.125 to ≥ 256 µg/mL), against six *P. aeruginosa* were determined using the broth microdilution method following the CLSI guidelines [45]. The final concentration of the isolates in Mueller-Hinton (MH) broth was 1.5 × 10^5^ CFU/mL in 96-well flat-bottom microtiter plates, and the serial concentrations of CHG were used (including 0, 0.125, 0.25, 0.5, 1, 2, 4, 8, 12, 16, 24, 32 and 64 µg/mL. The plates were incubated at 37℃ for 18 h. All the MIC tests were performed in triplicates. The MIC was defined as the minimum concentration of the drug that inhibited the visible growth of bacteria [46]. When the MIC of CHG against the *P. aeruginosa* strain was over 50 µg/mL, the strain was considered a CHG-tolerant strain [47, 48].

### Time-killing curves of bacteriophage with CHG

The bacteriostatic effects of the combination against six CHG-tolerant *P. aeruginosa* strains and reference strain PAO1 was determined in LB broth using 1 × 10^8^ PFU/mL bacteriophage vB3530 and sub-MICs of CHG (1/2×MIC, 1/4×MIC and 1/8×MIC). Bacterial suspensions were inoculated into sterilized tubes, containing 20 mL LB as described above.The experiment was divided into eight groups, including a bacteria control group without any treatment, three CHG treatment groups (1/2×MIC, 1/4×MIC, and 1/8×MIC), bacteriophage treatment group, and three phage/CHG combined treatment groups (1 × 10^8^ PFU/mL phage + 1/2×MIC CHG, 1 × 10^8^ PFU/mL phage + 1/4×MIC CHG, and 1 × 10^8^ PFU/mL phage + 1/8×MIC CHG). All the groups were cocultured with fresh 200 µL of 0.5 McFarland strain suspension. Then bacteriophage-treated groups were added with a final concentration of 10^8^ PFU/mL of bacteriophages. The CHG-treated groups were added with a final concentration of sub-MIC CHG. The combined treatment groups were added with CHG and bacteriophage. The tubes were incubated at 37℃ and 180 rpm. Finally, 100 µL of the test samples were collected for viable bacteria counts after 2, 4, 6, 12, and 24 h of incubation [49]. Each experiment was performed three times.

### Determination of the emergence rates of bacterial mutants

In order to determine the frequency of phage-resistant bacteria treated with bacteriophage only (no CHG), 10 isolated colonies were selected from a plate to incubate at 37℃ for 12 h to prepare LB bacterial cultures. Then, 10^− 1^ to 10^− 8^ dilutions of the bacterial cultures and phage were made (from a stock solution at 10^9^ PFU/mL), and 100-µL aliquots of the diluted solutions were inoculated in tubes containing 0.4% LB agar, plated on LB agar plates, and incubated at 37℃ for 48 h. Similarly, the frequency of CHG-tolerant strains treated with 1/2×MIC CHG only (no phage) was determined; 100 µL aliquots of 10^− 1^ to 10^− 8^ dilutions from bacterial cultures were plated on LB agar plates with CHG and incubated for 48 h at 37℃. Meanwhile, 100 µL aliquots 10^− 1^to 10^− 8^ of the bacterial cultures were co-cultured with phage on LB agar plates containing CHG (CHG + phage) using a double-layer agar method to determine the emergence of CHG-tolerant and phage-resistant strains after treatment with bacteriophage and CHG.

Finally, the frequency of strains was calculated by dividing the number of resistant bacteria (obtained from ten isolated colonies) by the total number of susceptible bacteria. The differences in the frequencies of resistant strains in the CHG-only, phage-only, and CHG + phage groups were identified using one-way ANOVA [50, 51].

### Assessment of medical device disinfection using CHG and bacteriophage

The disinfection effects of the CHG and bacteriophage combination on medical devices were tested using contaminated syringe needles. In brief, sterile needles were first exposed to 1 × 10^6^ CFU/mL suspensions of PAO1 strain for 1 h. The contaminated needles were then removed and allowed to dry for 1 h. Next, the disinfectant solution was divided into three groups: phage vB3530 group (1 × 10^8^ PFU/mL), 1/2×MIC CHG group, and 1 × 10^8^ PFU/mL phage and 1/2×MIC CHG combined group. Then, the exposed needles were placed in disinfection solutions for 2 h. For the control group, the exposed needles were treated with an equal volume of normal saline. Finally, bacteria on the surface of needles were taken out to fully elute for colony counting.

### Statistical analyses

All the data were expressed as mean ± standard deviation of at least three independent experiments. Statistical significance was determined using an independent two-tailed *t*-test and one-way ANOVA. The *P*-values of < 0.05, < 0.01, and < 0.001 were denoted by *, **, and ***, respectively, while insignificance was denoted by ns. GraphPad Prism 8.0 was used for the statistical analysis.

## Data Availability

The datasets generated and/or analysed during the current study are available in the GenBank repository, [Accession number OR075999].

## List of abbreviations

CHG: Chlorhexidine
PFU: Plaque forming units
RAST: Rapid Annotations using Subsystems Technology
FDA: Food and Drug Administration
MDR: Multidrug resistance
ARG: Antibiotic resistant gene
MIC: Minimum inhibitory concentrations. Sub-MICs:Sub-inhibitory concentrations
CARD: The Comprehensive Antibiotic Resistance Database
MALDI-TOF MS: Matrix-assisted laser desorption/ionization time of flight mass spectrometry
CLSI: Clinical and Laboratory Standards Institute
VFPB: Virulence Factor Database
EOP: Efficiency of Plating
CBA: Columbia blood agar
CAZ: Ceftazidime
IPM: Imipenem
CIP: Ciprofloxacin
GEN: Gentamicin
MH: Mueller-Hinton
CFUs: Colony forming units
